# Supplementary material for: Causal Relationship between Aging and Anorexia Nervosa: A White-Matter-Microstructure-Mediated Mendelian Randomization Analysis
Source: Biomedicines. 2024 Aug 16;12(8):1874. doi: 10.3390/biomedicines12081874 (PMC11351342; doi:10.3390/biomedicines12081874)
Supplement: Supplementary file 1 [file biomedicines-12-01874-s001.zip › Supplementary Figure/Figure S8/Leaveoneout plot of MR analysis for TL to ubm-b-1707.pdf]

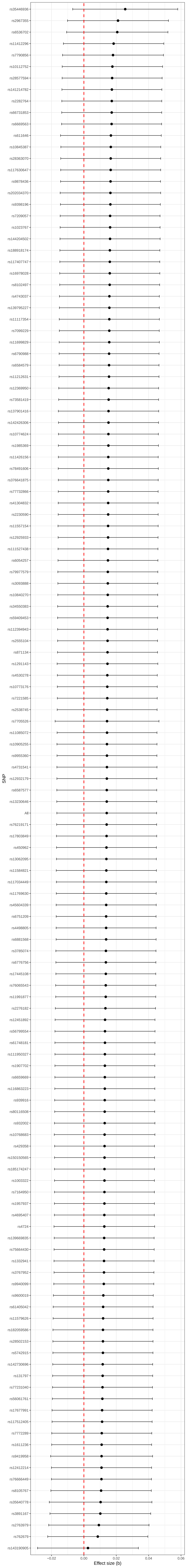

rs35446936
rs2967355
rs6536702
rs11412296
rs7790850
rs10112752
rs28577594
rs141214782
rs2282764
rs66731853
rs6669563
rs611646
rs10845387
rs28363070
rs117630647
rs9878436
rs202034370
rs9398196
rs7209057
rs1023767
rs144204502
rs188918174
rs117407747
rs16978028
rs8102497
rs4743037
rs139795227
rs11117354
rs7099229
rs11699829
rs6790988
rs6584579
rs11212631
rs12369950
rs73581419
rs137901416
rs142426306
rs10774624
rs1985369
rs11426156
rs78491606
rs376641875
rs77732866
rs41304832
rs2230590
rs11557154
rs12925933
rs111527438
rs6054257
rs79977579
rs3093888
rs10840270
rs34550383
rs59409453
rs112394943
rs2555104
rs871134
rs1291143
rs4530278
rs10773176
rs7221585
rs2538745
rs7705526
rs11085072
rs10905255
rs9955360
rs4731541
rs12932179
rs6587577
rs13230646
A8
rs76219171
rs17803849
rs450962
rs13062095
rs11584821
rs117034449
rs11769630
rs45604339
rs6751209
rs4498805
rs6881566
rs3785074
rs6776756
rs17445106
rs76065543
rs11991877
rs2276182
rs12451892
rs56799554
rs61748181
rs111950327
rs1907702
rs6659669
rs116863223
rs939916
rs80116508
rs932002
rs10768683
rs429358
rs150150565
rs185174247
rs1003322
rs7164950
rs1957937
rs4695407
rs4724
rs139669835
rs75664430
rs1332941
rs3767952
rs9940099
rs9600019
rs61405042
rs11579626
rs182059586
rs28502153
rs5742915
rs142730696
rs131797
rs77231040
rs56061761
rs17677991
rs117512405
rs7772289
rs1611236
rs9419958
rs12412214
rs76666449
rs8105767
rs35640778
rs3891167
rs2763979
rs762679
rs143190905
SNP
-0.02
0.00
0.02
0.04
0.06
Effect size (b)
